# Effect of temporal stimulus properties on the nociceptive detection probability using intra-epidermal electrical stimulation

**DOI:** 10.1007/s00221-015-4451-1

**Published:** 2015-10-05

**Authors:** Robert J. Doll, Annefloor C. A. Maten, Sjoerd P. G. Spaan, Peter H. Veltink, Jan R. Buitenweg

**Affiliations:** Biomedical Signals and Systems, MIRA Institute for Biomedical Technology and Technical Medicine, University of Twente, Zuidhorst, ZH-222, Drienerlolaan 5, PO BOX 217, Enschede, The Netherlands

**Keywords:** Nociception, Intra-epidermal electrical stimulation, Temporal stimulus properties, Psychophysics

## Abstract

Chronic pain disorders can be initiated and maintained by malfunctioning of one or several mechanisms underlying the nociceptive function. Although several quantitative sensory testing methods exist to characterize the nociceptive function, it remains difficult to distinguish the contributions of individual mechanisms. Intra-epidermal electrical stimulation of nociceptive fibers allows defining stimuli with temporal properties within the timescale of these mechanisms. Here, we studied the effect of stimulus properties on the psychophysical detection probability. A psychophysical detection experiment was conducted including 30 healthy human participants. Participants were presented with electrical stimuli having various temporal properties. The pulse-width was varied for single pulse stimuli (either 420 or 840 μs), and the inter-pulse interval for double pulse stimuli (10, 50, or 100 ms). Generalized linear mixed models were used to obtain estimates of thresholds and slopes of the psychophysical function. The 840-μs single pulse resulted in a lower threshold and steeper slope of the psychophysical function than the 420-μs single pulse. Moreover, a double-pulse stimulus resulted in a lower threshold and steeper slope than single pulse stimuli. The slopes were similar between the double pulse stimuli, but thresholds slightly increased with increasing inter-pulse intervals. In the present study, it was demonstrated that varying the temporal properties of intra-epidermal electrical stimuli results in variations in nociceptive processing. The estimated thresholds and slopes corresponding to the selection of temporal properties suggest that contributions of peripheral and central nociceptive mechanisms can be reflected in psychophysical functions.

## Introduction

Chronic pain disorders can be initiated and maintained by malfunctioning of one or several mechanisms underlying the nociceptive function. Prior to the perception of a nociceptive stimulus, the induced neural activity is processed by peripheral (Mendell 2011) and central mechanisms (Bromm and Lorenz 1998; Sandkühler 2009; Woolf 2011) which modulate the perceived strength and quality. Malfunctions of these mechanisms may lead to, for example, peripheral sensitization which is expressed by an increase in neural activity given the same stimulus strength. Another example is central sensitization which is expressed by a higher postsynaptic neural activity given the same incoming peripheral neural activity and is said to be an important factor in chronic pain disorders (Latremoliere and Woolf 2009).

Experimental application of stimuli and recording the responses allow the observation and quantification of nociceptive processing. Commonly used methods for observation of stimulus processing and sensory function include psychophysical quantitative sensory testing (QST) methods (Arendt-Nielsen and Yarnitsky 2009; Rolke et al. 2006). Multiple QST methods exist, using a broad range of stimulus types such as thermal, mechanical, or electrical. Additionally, QST can serve as a predictor of chronic pain development (Backonja et al. 2013; Wilder-Smith et al. 2010). It remains, however, difficult to distinguish the individual contributions of nociceptive mechanisms, hampering the diagnosis of chronic pain disorders.

For observing the contributions of individual nociceptive processes, stimuli must have properties such that specific characteristics of these processes are elicited. Recent studies have shown that intra-epidermal electrical stimulation can preferentially activate nociceptive Aδ fibers (Inui and Kakigi 2012; Kodaira et al. 2014; Mouraux et al. 2010), as long as the stimulation current is below twice the detection threshold in order to minimize the co-activation of tactile Aβ fibers (Legrain and Mouraux 2013; Mouraux et al. 2010). This limitation becomes problematic when estimating pain thresholds and pain tolerance thresholds. However, when presenting stimuli with varying amplitudes below this limitation, and recording the corresponding detections (i.e., detected or not-detected), the nociceptive detection probability, in terms of the threshold and slope of a psychophysical curve (Klein 2001; Treutwein 1995), can be safely estimated.

An advantage of electrical stimulation is the accurate control of stimulation timing allowing well-defined stimuli with temporal resolutions in the order of tens of μs. Varying the temporal properties of rectangular-wave current stimuli [i.e., the pulse-width (PW), number of pulses (NoP), and inter-pulse interval (IPI)] could allow probing the nociceptive system in more detail. Peripheral phenomena, such as the strength–duration relationship (Rollman 1969; Weiss 1901), can be probed by varying the PW. Inhibitory and/or facilitatory processes, either at a peripheral level or at a central level, could possibly be probed by varying the NoP and IPI (Mouraux et al. 2014; van der Heide et al. 2009).

**Fig. 1:**
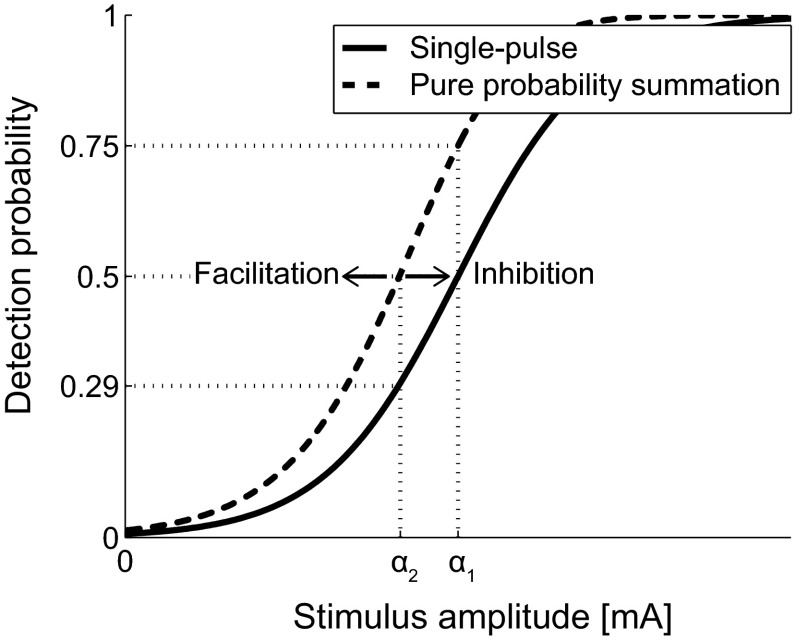
Effect of probability summation. The *thick line* presents the psychophysical curve for a single-pulse stimulus. The *thick dashed line* presents the psychophysical curve for a double-pulse stimulus, based on pure probability summation. An additional shift in the psychophysical curve can occur when the double-pulse stimulus is subject to inhibiting or facilitating processes

In the case of double pulse stimuli, it should be noted that the detection probability of a double-pulse stimulus depends on the detection probability of each of the individual pulses (*p*_1_ and *p*_2_, respectively) according to probability summation (e.g., Gescheider et al. 1999). The detection probability of a double-pulse stimulus can be formulated as *p*_d_ = 1 − (1 − *p*_1_)(1 − *p*_2_).When the separate detection probabilities of both pulses are independent and equal, this formula reduces to *p*_d_ = 1 − (1 − *p*_1_)^2^. We refer to the latter description as pure probability summation. Figure 1 illustrates the relation between the detection probability of a single-pulse stimulus and a double-pulse stimulus. Due to pure probability summation, the expected threshold for the double-pulse stimulus (*α*_2_) would equal the amplitude resulting in a 0.29 detection probability for a single pulse. Similarly, the threshold for a single-pulse stimulus (*α*_1_) is equal to the amplitude resulting in a 0.75 detection probability. Moreover, the psychophysical function for double pulse stimuli is steeper than for single pulse stimuli. When both pulses cannot be considered independent (i.e., when the detection probability of the second pulse is altered by the presence of the first pulse, hence *p*_1_ ≠ *p*_2_), either inhibitory of facilitatory processes were activated, resulting in a psychophysical curve which is shifted from the curve described by pure probability summation.

Presenting subjects with a mixed sequence of stimuli with different predefined temporal properties within a single experiment might allow the simultaneous observation of various contributions of nociceptive processes to stimulus processing. From the combination of stimuli and corresponding responses (i.e., detected or undetected), the detection probability per stimulus type can be described by a psychophysical function in terms of a threshold and a slope (Klein 2001; Treutwein 1995). While the threshold is often used to indicate altered nociceptive function [e.g., hyperalgesia (Treede et al. 1992)], the slope of the psychophysical function is not so often used but could provide additional information about the reliability of stimulus detection by participants (Gold and Ding 2013).

While the effect of temporal stimulus parameters, such as the PW and the NoP, have been studied before on the strength and quality of perception of pain, the effect on the detection probability has not been studied before. An experiment was performed where subjects were presented with stimuli with different temporal properties using intra-epidermal electrical stimulation. We study whether different temporal properties affect the psychophysical function, and whether changes in the psychophysical function reflect contributions of nociceptive processes.

## Methods

### Participants

The effect of stimulus properties on the nociceptive detection probability was studied in two psychophysical experiments, each including 15 pain-free human participants (14 men, 16 women; mean age = 22.0, SD = 1.6). The Medical Ethics Committee Twente approved all experiment procedures. All participants provided written informed consent and were rewarded with a gift voucher after participation in the experiment.

### Experiment design

**Fig. 2:**
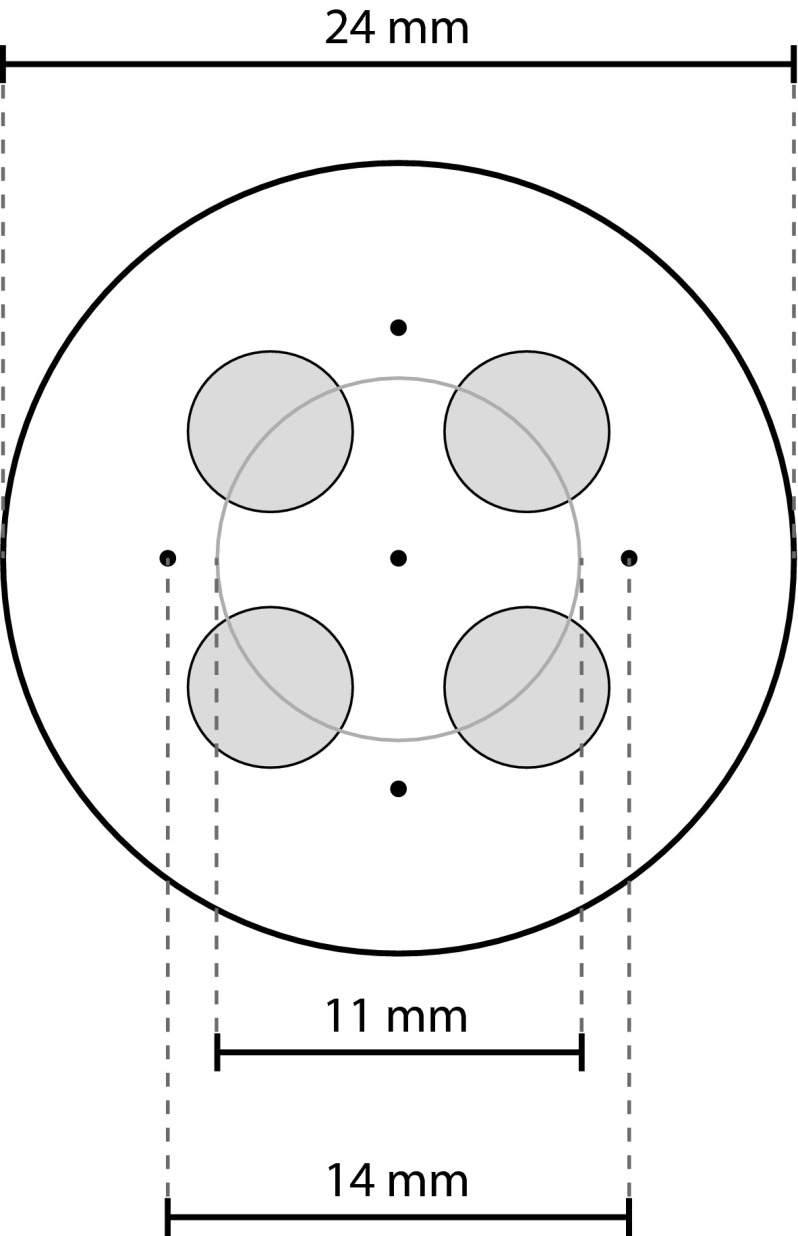
Schematic presentation of the needle electrode. The electrode consists of four interconnected 5-mm-diameter disk electrodes and five interconnected needle electrodes

Two identical psychophysical experiments were performed. Participants visited the laboratory on two consecutive days. All procedures on the first day were repeated on the second day. An electrode was attached to the participants’ left forearm which consisted of an array of five interconnected needles and four flat interconnected electrodes with a diameter of 5 mm [see Fig. 2 for a schematic representation, see (Steenbergen et al. 2012) for more details]. Prior to the experiment, participants were familiarized with the test procedures by presenting electrical stimuli with various amplitudes. Then, the 10-min experiment started in which test-stimuli were presented to participants.

### Test-stimuli

Cathodic rectangular pulses were applied as test-stimuli using four different settings of temporal properties (Table 1). In the first experiment, the electrode was covered with a conducting pad that covered the flat electrodes and served as the anode. For technical reasons, a 50 × 90 mm TENS electrode served as anode in the second experiment.

**Table 1:** Pulse-width (PW), number of pulses (NoP), and inter-pulse interval (IPI) used in both experiments

	Experiment 1			Experiment 2		
	PW (μs)	NoP	IPI (ms)	PW (μs)	NoP	IPI (ms)
Setting 1	420	1	–	420	1	–
Setting 2	840	1	–	420	2	10
Setting 3	420	2	10	420	2	50
Setting 4	420	2	50	420	2	100

Four different combinations of temporal electrical properties were randomly presented during the experiment. The combinations of temporal properties were different for both experiments. For the first experiment, the PW, NoP, and IPI were varied, and in the second experiment the NoP and IPI. The electrical properties of the stimuli used in the first and second experiments are presented in Table 1.

Stimulus amplitudes were selected according to an adaptive probing procedure (Doll et al. 2014). The procedure started with a predefined set of five equidistant stimulus amplitudes between 0 and 0.4 mA for single pulse stimuli and between 0 and 0.2 mA for double pulse stimuli. The amplitude of the upcoming stimulus was randomly selected from this set. All amplitudes in the set were increased and decreased with a fixed step size after a not-detected stimulus and detected stimulus, respectively. The step size was 0.1 mA for single pulse stimuli and 0.05 mA for double pulse stimuli. The different stimulus settings were presented in a randomly intermingled sequence.

Participants were instructed to indicate whether they detected a stimulus by releasing a response button and to press the button again after about a second. Due to this, the inter-stimulus interval after a detected stimulus was longer than after a not-detected stimulus. Besides the fixed inter-stimulus interval, an extra random interval between 0.6 and 1 s was added to reduce the stimulation predictability. This resulted in a mean inter-stimulus interval of 2.7 and 3.5 s after a not-detected stimulus and after a detected stimulus, respectively. A custom computer program (written in LabVIEW 2011, SP1) controlled all stimulation procedures, as well as the registration of stimulus amplitudes in mA, stimulation times in milliseconds, and responses to stimuli (i.e., detected or not-detected).

### Statistical analysis

All data preparation was performed in MATLAB 8.1 (MathWorks, Inc., Natick, Massachusetts, USA). Statistical modeling was performed using the lme4 library (Bates et al. 2014) in the R software package (R Core Team 2014). For each of the two experiments, a generalized linear mixed model (GLMM) using a logit link function was built to estimate the detection probability given the stimulus amplitudes. The intercept, stimulus amplitude [mA], study day, setting, stimulation time [s], and the interaction between amplitude and setting were included as fixed effects. Between-subjects random effects were included for the intercept, stimulus amplitude, study day, setting, and stimulation time. An unstructured covariance matrix was used to model the random effects. The stimulation time variable was centered and scaled prior to analysis to speed up the estimation process.

Type III Wald Chi-square statistics were used to test the main and interaction effects of the fixed effects. Confidence intervals of the regression parameters were based on the Wald z statistics. Threshold estimates were obtained from the regression parameters, and corresponding standard errors were approximated using the Delta procedure (Faraggi et al. 2003; Moscatelli et al. 2012). The slope estimates were obtained from the regression parameters and are equal to the estimated log-odds of the stimulus amplitude and corresponding interactions with setting. Post hoc multiple comparisons between thresholds and slopes were performed using Bonferroni *p* value corrections.

## Results

A total of 30 participants divided over two experiments participated in the experiment. The second day data of two participants in the first experiment, as well as data of one participant for both study days in the second experiment, were excluded from the analysis due to technical issues. About 50 stimulus response pairs (SRP) (mean = 49.6, SD = 4.2) were available per participant, per setting, per study day. Therefore, participants were presented with about 400 stimuli in total.

Table 2 presents the results of the two GLMM models. For both experiments, the intercept, stimulus amplitude, stimulation time, and the interaction between setting and stimulus amplitude as fixed effects significantly affected to the detection probability. While insignificant for the first experiment, setting had a significant effect on the detection probability in the second experiment. Moreover, the detection probability was similar on both study days.

**Table 2:** Type III Wald statistics

Parameter	Experiment 1		Experiment 2	
	*χ* ^2^(*df*)	*p*	*χ* ^2^(*df*)	*p*
(Intercept)	78.3 (1)	<.001	41.8 (1)	<.001
Stimulus amplitude	81.5 (1)	<.001	17.9 (1)	<.001
Setting	1.4 (3)	.705	26.7 (3)	<.001
Stimulation time	24.4 (1)	<.001	34.7 (1)	<.001
Study day	0.2 (1)	.640	0.1 (1)	.791
Setting × stimulus amplitude	106.1 (3)	<.001	49.1 (3)	<.001

**Fig. 3:**
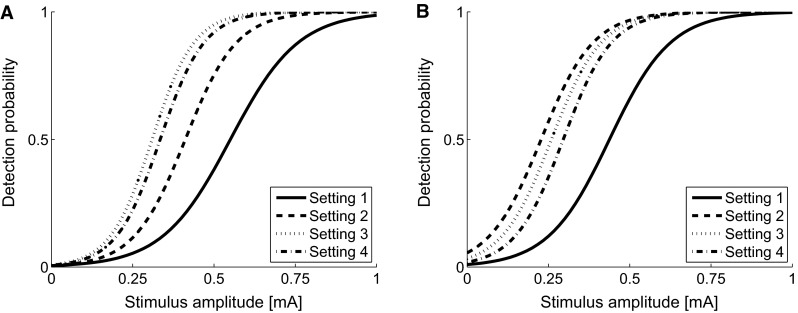
Psychophysical curves for both experiments for each stimulus setting (Table 1). The curves are obtained from the regression parameters (Table 3)

The estimated log-odds for the regression parameters and corresponding 95 % confidence intervals are presented in Table 3. Note that the stimulation time variable was transformed using a z-transformation prior to the analysis. Therefore, all obtained parameters reflect the estimated parameters at the middle of the experiment. The effect of time on the psychophysical curve ranged from 0.01 to 0.02 mA/min and was similar in both experiments. The regression parameters were inverse-logit transformed to obtain the logistic psychophysical curves for all settings at the first study day (Fig. 3).

**Table 3:** Regression parameter estimates of the fixed effects and corresponding confidence intervals

Parameter	Experiment 1		Experiment 2	
	Estimate	95 % Confidence interval	Estimate	95 % Confidence interval
(Intercept)	−5.25	[−6.41 −4.09]	−4.63	[−6.03 −3.23]
Stimulus amplitude	9.54	[7.47 11.61]	10.58	[5.68 15.49]
Setting				
Setting 2	0.05	[−0.60 0.71]	1.80	[1.01 2.60]
Setting 3	0.47	[−0.38 1.33]	1.26	[0.56 1.96]
Setting 4	0.32	[−0.53 1.17]	0.67	[−0.05 1.39]
Stimulation time	−0.52	[−0.73 −0.32]	−0.68	[−0.91 −0.46]
Study day	−0.34	[−1.77 1.09]	0.12	[−0.76 1.00]
Setting × stimulus amplitude				
Setting 2	3.09	[1.98 4.20]	1.86	[0.99 2.72]
Setting 3	5.90	[4.55 7.25]	2.42	[1.49 3.36]
Setting 4	5.20	[3.92 6.47]	2.82	[1.88 3.75]

Presented values are the log-odds

**Fig. 4:**
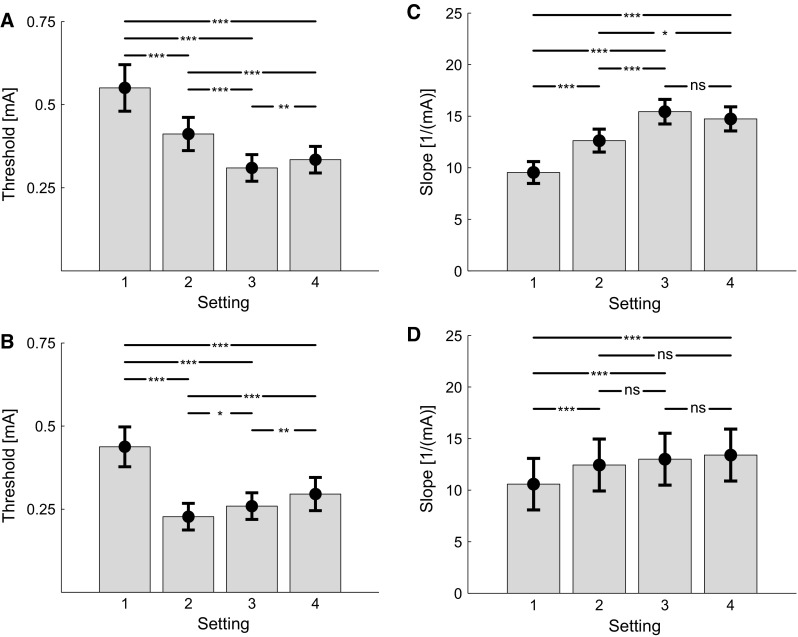
Estimated thresholds (**a**, **b**) and slopes (**c**, **d**) and corresponding standard errors for both experiments for each stimulus setting (Table 1). Overall, increasing the pulse-width and number of pulses resulted in a decrease in detection threshold and in an increase in the slope. Increasing the number of pulses results in an increased detection threshold, but no observable difference in the slope. *, **, and *** indicate a significant mean difference with a value of *p* < .05, *p* < .01, and *p* < .001, respectively

The estimated thresholds and slopes in both experiments are presented in Fig. 4. Post hoc comparisons between settings (Table 1) showed that increasing the PW from 420 to 840 μs for a single pulse decreased the threshold and increased the slope (Fig. 4a, c). Increasing the NoP from 1 to 2 pulses resulted in a decrease in threshold and increase in slope as well (Fig. 4a–d). Moreover, increasing the IPI between two consecutive pulses slightly increased the threshold, but did not significantly affect the slope.

## Discussion

We performed two psychophysical experiments, each including 15 human participants, to study the effect of temporal stimulus properties on the nociceptive detection probability. Participants were presented stimuli with four different combinations of temporal properties. Temporal properties in the first experiment were chosen such that the PW, NoP, and IPI were varied. The properties in the second experiment were chosen to put more emphasize on the NoP and IPI. We studied whether the different temporal stimulus properties affect the detection probability in terms of the threshold and slope and whether differences reflect contributions of nociceptive processes.

A needle electrode (Steenbergen et al. 2012) was used for intra-epidermal electrical stimulation. Recent studies have shown that this type of stimulation device allows the preferential stimulation of nociceptive Aδ fibers, provided that the amplitudes of stimuli are below twice the detection threshold (Legrain and Mouraux 2013; Mouraux et al. 2010). In the present experiment, stimulus amplitudes were chosen according to an adaptive stimulus selection procedure (Doll et al. 2014, 2015) such that the amplitudes were always near the detection threshold. Some of the presented single pulse stimuli might have had amplitudes near twice the estimated detection threshold and possibly co-activated Aβ fibers. Even if these activated fibers would have promoted the detection probability of these stimuli, the corresponding SRPs would have a small influence on the estimation of the detection threshold, as these SRPs were not near the detection threshold. Hence, the detection of a stimulus is likely to be preferentially mediated by activation of Aδ fibers, without substantial contribution of co-activated Aβ fibers. To further explore the contributions of Aβ fibers, future studies could include evoked potentials to demonstrate the preferential stimulation of nociceptive fibers (e.g., see Mouraux et al. 2010, 2014; van der Heide et al. 2009).

The detection thresholds found in this study are higher than those presented by Otsuru et al. (2010) and Mouraux et al. (2010), but similar to those previously observed in our group (Doll et al. 2014; Steenbergen et al. 2012). The design of the electrode is the biggest difference between these studies. While Otsuru et al. (2010) and Mouraux et al. (2010) used a single-needle electrode, we used a compound electrode consisting of five needles (Fig. 2). As the needles were interconnected, the total current is, assuming ideal needles and with similar electrical impedances, evenly distributed over the five needles. As a result, the total current necessary for neural activation is about five times higher for a five-needle electrode than for a single-needle electrode. Therefore, the thresholds presented in this paper should be divided by five for a better comparison. Doing so would show that the thresholds are then in a similar range as those presented by Otsuru et al. (2010) and Mouraux et al. (2010).

Three sets of temporal settings in the first experiment were also used in the second experiment (see Table 1), and thus similar results were expected. However, the estimated thresholds in the first experiment are higher than those observed in the second experiment. The difference between the two experiments was the electrode configuration. A conducting pad covering the flat electrodes was used as anode in the first experiment, but was not used in the second experiment. This slightly increased the length of the needles and could possibly decrease the distance between needles and nerve fibers. Moreover, the location of the anode in the second experiment was further away from the needles than the conducting pad was. Therefore, a difference in neuro-electrical interface was present between the first and second experiments, likely explaining the difference in thresholds for the same temporal properties. However, this does not affect the overall conclusions of this paper.

When comparing the estimated thresholds of the two single pulse stimuli with different PW, a higher detection threshold was observed when the PW was 420 µs than when the PW was 840 µs. The threshold found for the shorter PW was, on average, 0.55 mA in the first experiment. This indicates that a total charge of about 420 µs × 0.55 mA = 0.23 µC was necessary for detection. Given a mean threshold of about 0.41 mA for the longer pulse stimulus, a charge of about 0.34 µC was necessary for detection. This charge is about 1.5 times higher than the charge for the shorter pulse stimulus. Therefore, increasing the PW results in an increase in charge necessary to elicit a detection. This phenomenon is related to the well-known strength–duration relationship describing the minimum charge required for peripheral activation (Geddes 2004; Rollman 1969). Given the estimated thresholds, the chronaxie value was calculated as 435 μs. While chronaxie values are dependent on several external factors making estimates difficult to interpret (Geddes 2004), the calculated value here is relatively high, supporting the argument of preferentially stimulating Aδ fibers (Li and Bak 1976; West and Wolstencroft 1983).

**Fig. 5:**
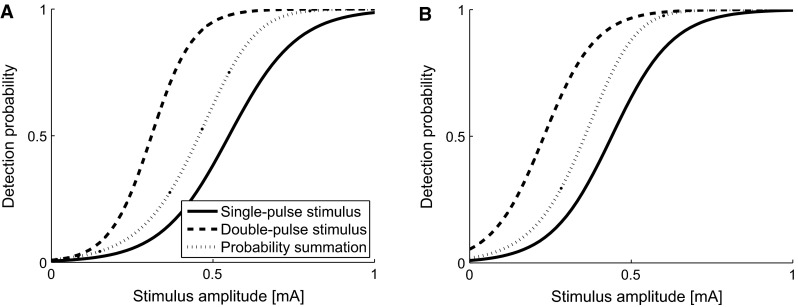
Effect of double pulse stimulation. **a** Presents data from the first experiment, and **b** from the second experiment. Based on probability summation, a decrease in threshold and increase in slope are expected for double pulse stimuli. However, the estimated curves for double pulse stimuli show even lower thresholds, and slightly steeper slopes

When the number of pulses is increased in a stimulus, a decrease in threshold and an increase in slope are expected based on pure probability summation (Fig. 1). Using the regression parameters found for the single-pulse stimulus (Table 3), the expected psychophysical curve for double pulse stimuli can be calculated. Figure 5 shows the psychophysical curves for single pulse stimuli, double pulse stimuli, and the curve based on pure probability summation. In both experiments, the estimated thresholds for double pulse stimuli are clearly lower than the expected thresholds based on pure probability summation. This suggests that pure probability summation does not fully account for a decrease in the threshold when the NoP is increased, and thus the detection probability of the second pulse is facilitated by the first pulse. Candidate mechanisms explaining this facilitation might reside at the peripheral as well as at the central level and both possibilities are considered below.

When considering the possibility of a peripheral facilitatory mechanism, it should be noted that a single stimulus pulse can activate a certain number of neural fibers in the skin, generating a certain amount of neural activity. The number of activated fibers and hence the amount of neural activity will increase with the pulse amplitude (and PW), and this amount determines the probability that the pulse is detected by the subject. Hence, if the detection probability of the second pulse in a double-pulse stimulus is increased by a peripheral mechanism, then the second pulse must generate a larger amount of neural activity than the first pulse, most likely by activating an additional number of fibers. As these additional fibers were not activated by the first pulse in the double-pulse stimulus, the presence of this pulse must have caused a change in the excitability of these fibers. This phenomenon is known as subthreshold superexcitability and is demonstrated in human experiments (Bostock et al. 2005; Shefner et al. 1996). In these studies, motor axons were found to be superexcitable up to about 30 ms after a near-threshold depolarizing pulse. As far as we are aware, no such effects for IPIs longer than 30 ms were reported. Also, we did not find reports of such effects specifically for cutaneous nociceptive fibers. In our study, we observed facilitation for IPIs ranging between 10 and 100 ms. Although this range exceeds the IPIs reported in literature, subthreshold superexcitability effects may still have occurred, as cutaneous nociceptive fibers have a much smaller diameter and might have longer time constants.

One of the possible central mechanisms leading to facilitation of the second pulse by the first is temporal summation of postsynaptic potentials. Another possibility is short-term synaptic plasticity (Zucker and Regehr 2002), such as paired-pulse facilitation or augmentation, enhancing the postsynaptic response after the second pulse which follows the first pulse. This effect was observed in synapses at several locations in the nociceptive system for IPIs ranging between tens and hundreds of ms (Luo et al. 2014). Both temporal summation and short-term plasticity can result in facilitation of the second pulse, but no distinction between the two can be made at this point.

With the current observations, peripheral and central contributions cannot be distinguished and future studies could focus on characterizing peripheral contributions. A first step would be to study subthreshold superexcitability of nociceptive axons. Another option which helps separating peripheral from central contributions is recording the membrane potentials using microneurography.

## Conclusion and outlook

In the present study, it was demonstrated that varying the temporal properties of intra-epidermal electrical stimuli results in variations in nociceptive processing. The estimated thresholds and slopes corresponding to the selection of temporal properties suggest that contributions of (a combination of) peripheral and central nociceptive mechanisms can be reflected in psychophysical functions. The stimulation paradigm has the potential of being of additional value to currently used neurophysiological and psychophysical measurements for quantification of the current state of nociceptive function. We argue that there is a need of further exploration of behavior of underlying mechanisms using electrical stimuli. Pharmacological interventions which selectively affect peripheral (e.g., application of capsaicin) and/or central mechanisms (e.g., (s)-ketamine) can be used for validation of the responsiveness of the presented methodology. Moreover, combining the presented methodology with computational models of nociceptive mechanisms has the potential of exploiting the newly gained observability of the nociceptive system using a mechanism-based interpretation (e.g., based on a system identification approach).
